# Pulmonary capillary pressure in pulmonary hypertension

**DOI:** 10.1186/cc3038

**Published:** 2005-02-11

**Authors:** Rogerio Souza, Marcelo Britto Passos Amato, Sergio Eduardo Demarzo, Daniel Deheinzelin, Carmen Silvia Valente Barbas, Guilherme Paula Pinto Schettino, Carlos Roberto Ribeiro Carvalho

**Affiliations:** 1 Pulmonary Division, Respiratory ICU – Heart Institute (InCor), University of São Paulo Medical School, São Paulo, Brazil

## Abstract

**Introduction:**

Pulmonary capillary pressure (PCP), together with the time constants of the various vascular compartments, define the dynamics of the pulmonary vascular system. Our objective in the present study was to estimate PCPs and time constants of the vascular system in patients with idiopathic pulmonary arterial hypertension (IPAH), and compare them with these measures in patients with acute respiratory distress syndrome (ARDS).

**Methods:**

We conducted the study in two groups of patients with pulmonary hypertension: 12 patients with IPAH and 11 with ARDS. Four methods were used to estimate the PCP based on monoexponential and biexponential fitting of pulmonary artery pressure decay curves.

**Results:**

PCPs in the IPAH group were considerably greater than those in the ARDS group. The PCPs measured using the four methods also differed significantly, suggesting that each method measures the pressure at a different site in the pulmonary circulation. The time constant for the slow component of the biexponential fit in the IPAH group was significantly longer than that in the ARDS group.

**Conclusion:**

The PCP in IPAH patients is greater than normal but methodological limitations related to the occlusion technique may limit interpretation of these data in isolation. Different disease processes may result in different times for arterial emptying, with resulting implications for the methods available for estimating PCP.

## Introduction

Pulmonary capillary pressure (PCP) is the major force determining fluid filtration from pulmonary capillaries into the interstitium, and thus it is the major determinant of oedema formation [1,2]. Measurement of PCP is therefore of clinical importance. However, equally important is the methodological difficulty in measuring it. Many methods for estimating PCP have been described, including the Gaar equation [1], the osmometric method [3] and others [4-7]. Because of the inaccuracy of the Gaar equation and because the other methods are not suitable for clinical application, pulmonary artery occlusion is currently the most frequently used method for estimating PCP in a broad range of clinical and/or experimental conditions [8].

The pulmonary artery occlusion method is based on the assumption that one can determine the pulmonary capillaries' emptying pattern from the decaying pulmonary arterial occlusion pressure waveform. However, this method does not allow reliable visualization of the two separate emptying phases of the arteries and capillaries in patients, and thus it obscures the point at which the capillary pressure should be derived. In order to obtain a better estimate of PCP, some investigators have represented the pulmonary circulation as an electrical circuit model and used various mathematical approaches to analyze the pressure decay after balloon occlusion. The complexity of the circuit reflects whether the initial decrease in the postocclusion pressure is linear or nonlinear.

The simplest circuit model includes an arterial resistor but no arterial capacitor. However, a three-compartment model proposed by Baconnier and coworkers [9] includes two resistors, representing arterial and venous pulmonary resistance, interposed among three capacitors in series. The latter represent arterial, capillary and venous capacitance compartments, respectively. Under the usual conditions, the capillary compartment would be the dominant capacitor whereas arterial resistance would be the major resistor.

The two primary mathematical approaches to analyzing the pressure decay after balloon occlusion consist of a monoexponential curve, which is fitted from 200 to 300 ms after the instant of occlusion, and a biexponential curve, which is fitted beginning at the instant of occlusion [10-14]. The monoexponential curve corresponds to the emptying of the capillary capacitance compartment, with only one time constant; the biexponential curve theoretically represents the complex emptying of two capacitance compartments (arterial plus capillary). Thus, the main difference between these two types of analysis is whether the arterial capacitance is computed. A useful feature of biexponential modelling is the opportunity to study emptying rates (time constants) of two compartments, which reflect the dynamics of the system and the relationship between capacitance and resistance downstream of each compartment.

Use of the pulmonary artery occlusion technique has identified high levels of PCP in patients with pulmonary arterial hypertension [15,16], even though lung oedema is not a feature of this clinical condition. However, no studies of the time constants of the various compartments of the pulmonary circulation in this condition have yet been conducted; such studies would clarify the mechanisms that lead to these PCP levels. The main objective of the present study was to compare estimates of PCP in patients with idiopathic pulmonary arterial hypertension (IPAH) obtained through different methods; all of these methods assumed the three-compartment model of pulmonary circulation, but each fits the pulmonary artery pressure (PAP) decay to the rather different algorithms proposed in the literature [8,11,17]. Because a 'gold standard' for PCP measurement at the bedside does not exist, we compared estimates of PCP in patients with IPAH versus well described estimates of PCP in patients with acute respiratory distress syndrome (ARDS) [18,19]. We hypothesized that, independent of the precise correspondence between the three-compartment model and the real pulmonary circulation, comparing the emptying rates of the capacitance territories in these two different pathological conditions should reveal important aspects of the pulmonary circulation.

## Methods

### Patients

We studied two groups of patients with pulmonary hypertension (defined as a mean PAP greater than 25 mmHg): patients with IPAH and patients with ARDS. All patients provided informed consent, and our institutional ethics committee approved the study protocol.

#### Idiopathic pulmonary arterial hypertension

Twelve patients with IPAH, according to the World Health Organization World Symposium definition [20], were included. All patients were breathing spontaneously. The data used in the present study were collected at the time of acute vasodilator test, before treatment with vasoactive drugs for IPAH.

#### Acute respiratory distress syndrome

Eleven patients diagnosed with ARDS, according to criteria defined by the American–European Consensus Conference on ARDS [21], were included. These patients had a mean PAP greater than 25 mmHg and were undergoing mechanical ventilation.

### Haemodynamic monitoring

A 7-F pulmonary artery catheter (Baxter Healthcare Corporation, Irvine, CA, USA) was introduced in all patients. The catheter position was verified by comparing the variation in pulmonary diastolic pressure during the respiratory cycle with the variation in wedge pressure at the same time [22]. Cardiac output was measured using the standard thermodilution technique.

The ports of each lumen were connected to transducers (HP1290C; Hewlett-Packard, Waltham, MA, USA) and pressure modules (M1006A; Hewlett-Packard) were connected to a Hewlett-Packard monitor (M1176-A; Hewlett-Packard). This monitor was previously modified with a continuous analogue/digital data output that allowed us to record the pressure curves on a personal computer, at 200 Hz. The acquisition and analysis of the decay curves were based on customized LabVIEW software (National Instruments, Austin, TX, USA).

### Ventilatory parameters

Haemodynamic data were collected in all patients under mechanical ventilation during a period of standard ventilation, with the following parameters/settings: pressure control ventilation, positive end-expiratory pressure 5 cmH_2_O, tidal volume 8 ml/kg, inspired fractional oxygen 1, inspiratory time 1 s, and respiratory rate 10 breaths/min. An expiratory pause of 10 s was imposed during data acquisition.

During data acquisition, patients breathing spontaneously were instructed to maintain a relaxed expiratory pause of at least 10 s in order to minimize artifacts caused by respiratory variations in the intrathoracic pressure.

### Pulmonary artery pressure curves

**Figure 1:**
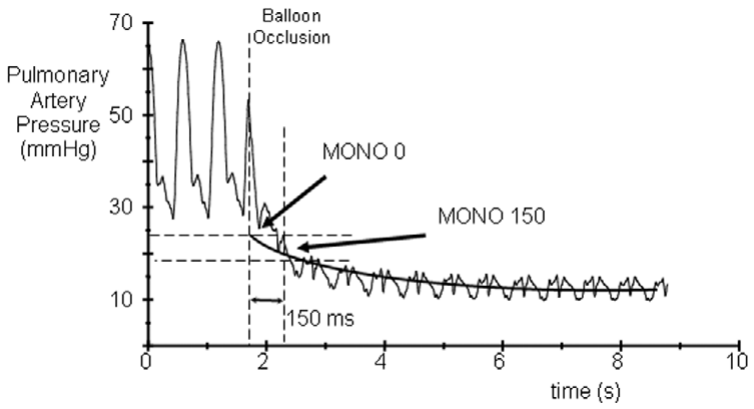
Monoexponential curve fitting for estimation of pulmonary capillary pressure (PCP). MONO 0, PCP obtained by extrapolation back to the time of occlusion; MONO 150, PCP obtained 150 ms after balloon occlusion.

**Figure 2:**
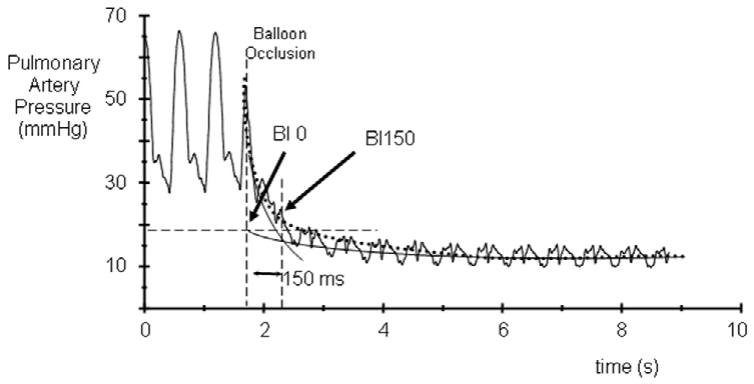
Biexponential curve fitting for estimation of pulmonary capillary pressure (PCP). BI 0, PCP obtained by extrapolation of the slow component back to the time of occlusion; BI 150, PCP obtained 150 ms after balloon occlusion.

In order to obtain two optimal curves, we obtained five PAP curves from each patient. We defined optimal curves to be those with at least 10 s without respiratory oscillations after balloon occlusion and with occurrence of balloon occlusion during the fast rising phase of ventricular systole; these parameters allowed us to determine the precise time of occlusion (Figs 1 and 2).

### Curve fitting

We used two methods (monoexponential and biexponential) for curve fitting. The monoexponential method considers only the situation at 200 ms after balloon occlusion, and then extrapolates the fit back to the time of balloon occlusion or to some other time point. In contrast, the biexponential method considers all time points following balloon occlusion. We used a customized routine for curve fitting, employing the algorithm proposed by Foss [23].

Wedge pressure was considered to be the average pressure obtained after a steady state of at least 1 s. Analysis of the fitted curve allowed us to calculate the time constant of pressure decay. We were able to calculate the single time constant for the monoexponential curve and the two time constants, for the fast and slow components, for the biexponential curve.

### Pulmonary capillary pressure algorithms

We used the four most commonly employed algorithms to represent the PCP (Figs 1 and 2): mono 0, the value obtained by extrapolating the monoexponential fit back to the time of balloon occlusion; mono 150, the value obtained by extrapolating the monoexponential fit back to 150 ms after balloon occlusion; BI 0, the value obtained by extrapolating the slow component of the biexponential fit back to the time of balloon occlusion; and BI 150, the value obtained 150 ms after balloon occlusion in the biexponential fitted curve.

### Statistical analysis

We used repeated measures analysis of variance (two way) to compare PCP values between the groups, and repeated measures analysis of variance (one way) to compare the other haemodynamic data between groups.

## Results

Pulmonary haemodynamic data for all the patients, as well as time constants calculated from both mathematical analyses, are shown in Table 1. Patients with IPAH had a significantly lower cardiac index. These results were expected because IPAH is frequently associated with severe cardiac dysfunction whereas ARDS is often associated with hyperdynamic states [24].

**Figure 3:**
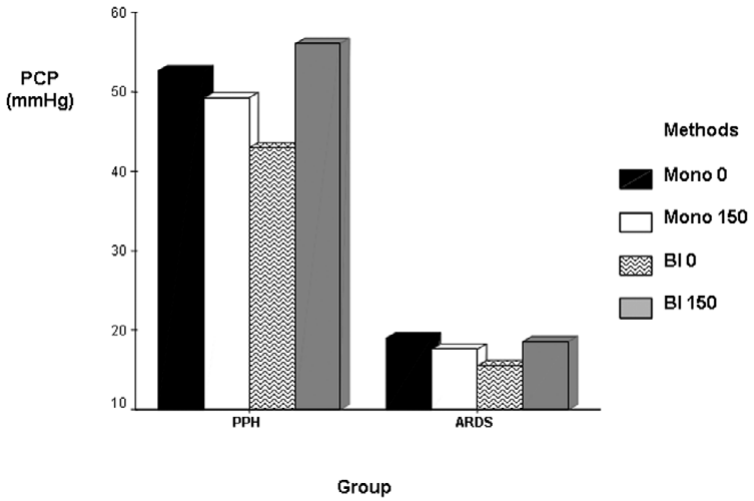
Estimates of pulmonary capillary pressure (PCP) using four different methods in patients with idiopathic pulmonary arterial hypertension (IPAH) and acute respiratory distress syndrome (ARDS). Significance for difference between IPAH and ARDS groups: *P *< 0.001. Significance for difference within groups: *P *< 0.02. BI 0: PCP obtained by extrapolation of the slow component back to the time of occlusion; BI 150, PCP obtained 150 ms after balloon occlusion; MONO 0: PCP obtained by extrapolation back to the time of occlusion; MONO 150: PCP obtained 150 ms after balloon occlusion.

Although patients with IPAH had a very high mean PAP, those with ARDS exhibited only mild pulmonary hypertension. The PCP values for both groups, estimated using all of the algorithms, are shown in Fig. 3. The PCP values in the IPAH group were significantly higher than those in the ARDS group (*P *< 0.001). The different algorithms also yielded PCP values that significantly differed within each group (*P *< 0.02).

**Figure 4:**
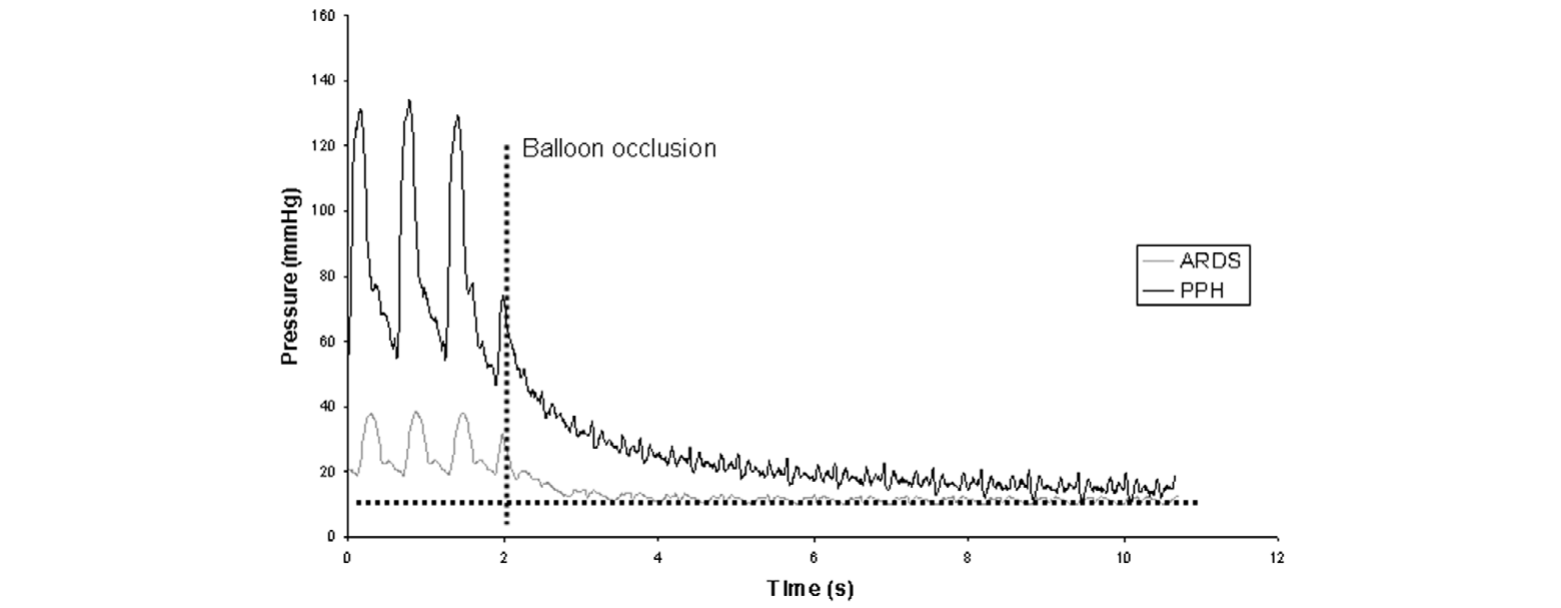
Pulmonary artery pressure decay curve in a patient with acute respiratory distress syndrome (ARDS; gray line) as compared with that in a patient with idiopathic pulmonary arterial hypertension (IPAH; black line). The time necessary to reach a steady state is longer in the patient with IPAH.

The time constant obtained from the monoexponential fit and the time constant obtained for the slow component of the biexponential fit were much higher in the IPAH than in the ARDS group (*P *< 0.001; Table 1). No difference between groups was found in the time constant for the fast component. Comparison of PAP decay curves from the two groups (Fig. 4) illustrates effect of these findings – the time required to reach a steady state was longer in IPAH group.

## Discussion

The major finding of the present study was that the IPAH group had a significantly higher PCP than did the ARDS group, independent of algorithm used for its estimation; this was accompanied by a marked increase in the time constant for the slow decay.

The PCP is a key determinant of the pathophysiology of the cardiopulmonary system. Commonly, the PCP is considered to be close to the wedge pressure; although this assumption is quite true under normal circumstances, it can lead to incorrect interpretations under pathological conditions [25]. In our study, in agreement with data previously reported by Kafi [15] and Fesler [16] and their colleagues, we found that the effective filtration pressure can be dramatically underestimated by this assumption. Those studies showed high levels of PCP (>30 mmHg) in patients with pulmonary hypertension.

Under acute conditions, PCP levels greater than 30 mmHg can lead to movement of fluid from the capillaries into the interstitium and alveolar spaces, once this pressure exceeds the blood oncotic pressure. However, under chronic conditions an increased lymphatic drainage capability can prevent PCP levels as high as 40 mmHg from causing lung oedema [26]. However, before the high levels of PCP found in our study may be considered valid, we must account for some methodological issues. Hakim and Kelly [27] suggested that the arterial occlusion technique measures the pressure in vessels with diameters that range from 50 to 900 μm, which usually encompass the major territory of the pulmonary blood volume. However, the algorithms used to estimate PCP in our two groups yielded significantly different PCP values in each group; this suggests that each method reflects the pressure at different arterial sites, or perhaps the pressure in territories with different vascular diameters.

Considering the same three-compartmental model, we used both monoexponential and biexponential fitting processes to analyze PCP. Some methodological approaches, such as use of the monoexponential curve fitting process starting 200 ms after occlusion and the use of back extrapolation (looking back at a sampling time corresponding to 150 ms after occlusion), were taken from previous work by Gilbert and Hakim [28]. Both procedures assume that the influence of the fast arteriolar emptying process is negligible after a few milliseconds, allowing study of the emptying of an almost isolated capillary. However, in testing the arterial occlusion method under vasodilating and vasoconstricting conditions, Pellett and coworkers [29] demonstrated that 150 ms was not the optimal time at which to determine PCP in dogs with intact lungs.

Another methodological issue that must be considered when using the arterial occlusion technique is determination of the time of balloon occlusion, because mathematical modelling is based on this time point being the start of curve fitting. In experimental models the double occlusion technique permits perfect recognition of the time of occlusion; however, at the bedside the precise time point at which balloon inflation takes place can only be identified by a clear modification in the PAP curve. In our analysis, we accepted only those curves in which a clear cut in the systolic limb of the PAP curve was identifiable. Holloway [30] and Nunes [31] and their coworkers identified a difference between the PCP estimated using this method and that estimated using superimposed occluded and nonoccluded curves. Although significant, this possible difference does not alter the interpretation of our data because of the magnitude of PCP levels and time constants found in our study.

The significant changes in microvascular dynamics observed in individual disease states mean that the optimal time for estimating the PCP may differ between them. This is supported by the finding of different time constants for the exponential decays in our patients with IPAH as compared with those with ARDS, especially considering the constants for the slowly emptying compartments. All of the fitting methods tested in the present study assumed three basic hypotheses: the fast emptying of arteriolar territories into the capillary territory occurs in a few milliseconds; the time constant for the slow decay (corresponding to capillary emptying) is several times longer than that for arterial emptying; and the dominant capacitance in the pulmonary circulation is the capillary network, which therefore corresponds to the slowest emptying observed after occlusion.

Nevertheless, in settings involving pulmonary arterial hypertension, many considerations oppose these basic hypotheses. The arteriolar territory is heterogeneously abnormal, and therefore longer periods may be required for complete emptying, which also changes the relation with capillary emptying. The capillary territory still has a large cross-sectional area but a shorter length and decreased compliance; consequently, it may not represent the dominant capacitance in the pulmonary circulation.

It is likely that none of the proposed sampling times derived from experimental studies is valid for studies in humans, which are mainly conducted in the setting of pulmonary arterial hypertension. Because of slower arterial emptying, the time needed to estimate the PCP should be longer. The time constants for the fast compartments estimated in the present study were around 0.25 s, suggesting that at least 0.75 s (for 95% emptying) should be allowed for this fast arteriolar emptying. As such, the increased PCP levels reported could reflect a precapillary territory pressure, leading to erroneous interpretation of the data. Probably an individual sample time, based on the fast compartment time constant, should be used for PCP estimation, but this requires confirmation in experimental models of pulmonary hypertension.

Biexponential curve fitting appears to be preferable in this setting because it does not assume any fixed time constant for the fast compartment, but estimates it instead. However, one of the basic requirements for realistic bicompartmental fitting is that there are two exponential decay processes with very different time constants, preferentially differing by an order of magnitude. We are not sure that this condition can be met in patients with IPAH or ARDS (Table 1).

With regard to the high PCP values found in our patients, we do not believe that artifacts caused by non-instantaneous occlusion could account for our results or for the variability in results achieved with different methods. According to a study conducted by Fesler and coworkers [16], these high PCP levels could be explained by increased venous resistance. There is increasing recognition of venous involvement in the pathophysiology of pulmonary hypertension. However, the methodological limitations described above do not allow validation of the assumption that increased venous resistance is the only cause of high PCP levels in patients with pulmonary hypertension.

## Conclusion

We conclude that analysis of PAP decay curves permits a better understanding of the pulmonary microvasculature. However, analysis of these curves' time constants suggests that the 150–200 ms allotted for fast arteriolar emptying may be insufficient under pathological conditions. Whereas the mean PCP measured using the artery occlusion technique was greater than normal in our patients with IPAH, the methodological limitations related to this technique may limit the interpretation of these data in isolation.

## Key messages

• 	PCP is elevated in IPAH, although its interpretation must take into account the methodological limitations of measurement using the arterial occlusion technique.

• 	The time constants of pulmonary artery emptying may differ according to the disease process.

• 	The time constants may be useful for increasing the accuracy of PCP measurement using the arterial occlusion technique.

## Abbreviations

ARDS = acute respiratory distress syndrome; IPAH = idiopathic pulmonary arterial hypertension; PAP = pulmonary artery pressure; PCP = pulmonary capillary pressure.

## Competing interests

The author(s) declare that they have no competing interests.

## Authors' contributions

RS conducted patient monitoring and data analysis, participated in statistical analysis and drafted the manuscript. MBPA conceived the study, and participated in its design and statistical analysis. SED conducted patient monitoring and data analysis. DD participated in the design of the study and statistical analysis, and helped to draft the manuscript. CSVB participated in data analysis. GPPS participated in study coordination and data analysis. CRRC participated in the design and coordination of the study, and helped to draft the manuscript. All authors read and approved the final manuscript.

## Figures and Tables

**Table 1:** Haemodynamic data

Group	*n*	CI (l/min per m^2^)	Pwedge (mmHg)	Mean PAP (mmHg)	PVRI (dyn·s/cm^5 ^per m^2^)	Tc mono	Tc bi-fast	Tc bi-slow
IPAH	12	1.60 ± 0.33*	15.1 ± 4.0	85.3 ± 19.2*	3588.4 ± 1115.1*	1.51 ± 0.23*	0.28 ± 0.05	2.15 ± 0.41*
ARDS	11	4.23 ± 0.94	12.5 ± 2.1	29.0 ± 3.9	307.4 ± 67.5	0.70 ± 0.32	0.25 ± 0.13	1.30 ± 0.43

Values are expressed as mean ± standard deviation. *P ≤ 0.001 – comparison between IPAH and ARDS patients. ARDS, acute respiratory distress syndrome; CI, cardiac index; IPAH, idiopathic pulmonary arterial hypertension; PAP, pulmonary artery pressure; PVRI, pulmonary vascular resistance index; Pwedge, pulmonary artery occlusion pressure; Tc bi fast, time constant of the fast component of the biexponential curve(s); Tc bi slow, time constant of the slow component of the biexponential curve(s); Tc mono, time constant of the monoexponential curve(s).
